# Epidemiology of diabetes and complications among adults in the Republic of Ireland 1998-2015: a systematic review and meta-analysis

**DOI:** 10.1186/s12889-016-2818-2

**Published:** 2016-02-09

**Authors:** Marsha L. Tracey, Michael Gilmartin, Kate O’Neill, Anthony P. Fitzgerald, Sheena M. McHugh, Claire M. Buckley, Ronan J. Canavan, Patricia M. Kearney

**Affiliations:** 1 Department of Epidemiology and Public Health, University College Cork, Western Gateway Building, Cork, Republic of Ireland; 2 Department of Medicine, Royal College of Surgeons, Dublin, Republic of Ireland; 3 Department of Public Health, Heath Service Executive (HSE) South, Cork, Republic of Ireland; 4 Department of Endocrinology, St. Vincent’s University Hospital, Dublin, Republic of Ireland

**Keywords:** Ireland, Prevalence, Trends, Diabetes, Microvascular, Macrovascular, Adults, Epidemiology

## Abstract

**Background:**

Accurate estimates of the burden of diabetes are essential for future planning and evaluation of services. In Ireland, there is no diabetes register and prevalence estimates vary. The aim of this review was to systematically identify and review studies reporting the prevalence of diabetes and complications among adults in Ireland between 1998 and 2015 and to examine trends in prevalence over time.

**Methods:**

A systematic literature search was carried out using PubMed and Embase. Diabetes prevalence estimates were pooled by random-effects meta-analysis. Poisson regression was carried out using data from four nationally representative studies to calculate prevalence rates of doctor diagnosed diabetes between 1998 and 2015 and was also used to assess whether the rate of doctor diagnosed diabetes changed over time.

**Results:**

Fifteen studies (eight diabetes prevalence and seven complication prevalence) were eligible for inclusion. In adults aged 18 years and over, the national prevalence of doctor diagnosed diabetes significantly increased from 2.2 % in 1998 to 5.2 % in 2015 (*p*_trend_ ≤ 0.001). The prevalence of diabetes complications ranged widely depending on study population and methodology used (6.5–25.2 % retinopathy; 3.2–32.0 % neuropathy; 2.5-5.2 % nephropathy).

**Conclusions:**

Between 1998 and 2015, there was a significant increase in the prevalence of doctor diagnosed diabetes among adults in Ireland. Trends in microvascular and macrovascular complications prevalence could not be examined due to heterogeneity between studies and the limited availability of data. Reliable baseline data are needed to monitor improvements in care over time at a national level. A comprehensive national diabetes register is urgently needed in Ireland.

**Electronic supplementary material:**

The online version of this article (doi:10.1186/s12889-016-2818-2) contains supplementary material, which is available to authorized users.

## Background

Diabetes is a serious global public health issue which has been described as the most challenging health problem in the 21^st^ century [1, 2]. Cases of diabetes have progressively increased worldwide; between 1980 and 2008 there was a two-fold increase in the number of adults with diabetes [3]. Type 2 diabetes is the main driver of the epidemic, accounting for approximately 90 % of all cases [2]. The increasing burden of diabetes is driven primarily by rising levels of obesity and an ageing population [2, 4]. To date there is no national surveillance programme, or national population-based survey of diabetes in Ireland. Therefore it is difficult to quantify or monitor the impact of diabetes at a national level. Estimates from the International Diabetes Federation (2013) suggest that the prevalence of diabetes is in line with global trends. In 2000, the IDF estimated that the prevalence of diabetes was 3.2 % [5], this had increased to 6.5 % in 2013 [2].

Diabetes places a significant burden of care on the individual, health care professionals and the wider health system [1, 6]. Individuals with diabetes are two to four times more likely to develop cardiovascular disease relative to the general population and have a two to five-fold greater risk of dying from these conditions [7, 8]. Diabetes is a significant cause of blindness in adults, non-traumatic lower limb amputations and end-stage renal disease resulting in transplantation and dialysis [2].

Understanding the epidemiology of diabetes is essential to identify public health priorities. Accurate estimates of the burden of diabetes are essential for future planning and evaluation of services. While the IDF provides prevalence estimates for countries and regions, there are substantial variations in time trends as estimates are based on imputations [9, 10]. To date, estimates of diabetes prevalence in Ireland have been largely based on data from the 2007 National Survey of Health and Lifestyles in Ireland (SLÁN) [11]. Country specific prevalence rates have also been reported in the grey literature [2]; however these estimates have been extrapolated using data from the UK. The Euro Diabetes Index (2014) stated that there was a lack of reliable data to monitor diabetes related complications in Ireland [12]. To date, a comprehensive overview of the diabetes situation in Ireland has not been carried out. Therefore the rationale for carrying out this systematic review is to provide a comprehensive understanding of the diabetes situation in Ireland and to highlight current gaps in existing knowledge to inform future research. The aims of this review are (1) to systematically identify and summarise studies describing the prevalence of diabetes and the most common microvascular (retinopathy, neuropathy and nephropathy) and macrovascular complications among adults in Ireland between 1998 and 2014; and (2) to explore trends in diagnosed diabetes prevalence between 1998 and 2015.

## Methods

This review was produced according to Preferred Reporting Items for Systematic reviews and Meta-Analyses (PRISMA) guidelines for systematic reviews and meta-analyses [13]. Key words and study eligibility criteria were determined a priori.

### Search strategy

Both peer-reviewed journal articles and reports were considered for this review. A systematic literature search was carried out in PubMed and Embase databases to identify relevant studies reporting the prevalence of diabetes, microvascular or macrovascular complications among adults within the Republic of Ireland. Keywords and Medical Subject Headings (MeSH) terms included Ireland, prevalence, diabetes, microvascular, retinopathy, neuropathy, nephropathy, macrovascular and cardiovascular disease. Keywords were combined using the AND or OR operators (Additional file 1). Titles and abstracts of the resulting literature were screened for further consideration. Reference lists of articles were also examined to identify potentially relevant studies. In addition, a Google search was conducted using the keywords prevalence, diabetes, retinopathy, neuropathy, nephropathy and Ireland to identify relevant grey literature. Searches were carried out between January 2014 and March 2014. A second search was carried out in December 2015 to ensure the review included all up to date relevant information.

### Inclusion criteria

Studies were eligible for inclusion if they met the following criteria: (1) conducted in the Republic of Ireland between 1998 and 2014; (2) cross-sectional study design or baseline data from longitudinal studies; (3) prevalence estimates reported for adults aged ≥ 18 years, including men and women; (4) data provided on diabetes prevalence (including a self-report of a previous doctor diagnosis and undiagnosed diabetes) and/or the prevalence of microvascular complications (retinopathy, neuropathy, nephropathy) or macrovascular complications (myocardial infarction, congestive heart failure, stroke or TIA) in persons with diabetes; (5) if prevalence data were not reported, sufficient detail to calculate the numerator and denominator was provided; (6) the total sample size was ≥ 200; (7) adequate information was reported on the methods used.

### Exclusion criteria

Studies containing participants from Northern Ireland, restricted to a specific sub-population (including hospital-based studies), solely focused on type 1 diabetes, pre-diabetes or gestational diabetes were excluded from this review. Model estimates of prevalence were also excluded. If multiple articles provided information on a single study, the article detailing the most comprehensive data was selected. Full text articles were retrieved for all potentially eligible studies and were independently reviewed by three authors (MT, MG, and KON).

### Data abstraction and quality assessment

For each eligible study, three reviewers (MT, MG, and KON) individually collected relevant information using a structured data extraction form. The methodological quality of each included study was assessed using a critical appraisal checklist for studies used in systematic reviews addressing questions of prevalence [14]. This appraisal tool was developed to specifically examine the internal and external validity of prevalence data included in systematic reviews. Methodological quality was considered ‘low’ if three or less criteria were met, ‘moderate’ if four to six criteria were met and ‘high’ if seven to nine criteria were met. Articles were not excluded on the basis of quality. Any inconsistencies in data abstraction and quality assessment between reviewers were resolved through consensus.

### Statistical analysis

A meta-analysis was carried out using STATA version 13.1 (StataCorp, College Station, TX, USA). Studies were grouped into four categories: diagnosed diabetes among adults aged 18+ years; diagnosed and undiagnosed diabetes among adults aged 45+ years; diagnosed diabetes among adults aged 45+ years; undiagnosed diabetes among adults aged 45+ years. Pooled estimates of diabetes prevalence and 95 % confidence intervals (95 % CI) were calculated. Trends in pooled prevalence could not be explored as there was a lack of available data from different time points; therefore an overall estimate was provided for each group. Heterogeneity between studies was assessed by the Chi-square based Q test and I^2^ statistic. Potential publication bias was evaluated by the Begg’s test. A two-tailed *p <*0.05 was regarded to be statistically significant. High heterogeneity was found among studies reporting diabetes prevalence (I^2^ ≥ 75 %, p-value < 0.01) hence, pooled estimates were calculated using random-effects model using the method of DerSimonian and Laird [15]. The results from the meta-analysis were presented in a forest plot. To determine the robustness of the results, a sensitivity analysis, based on high quality studies, was carried out. A meta-analysis of the prevalence of diabetes complications was inappropriate; factors which influence prevalence estimates (e.g. time since diabetes diagnosis, type of diabetes, method of diagnosis) either varied between studies or were not reported. Instead a narrative synthesis provides a summary of relevant data.

#### Trends in diagnosed diabetes

As trends in diabetes prevalence could not be calculated by meta-analysis, original datasets from four national population based studies [16–19], identified during the literature search were obtained and analysed. In each dataset, diabetes was defined by a self-report of a previous doctor diagnosis. A detailed description on study methodology can be found elsewhere [18, 20]. Using data from these national surveys, multivariate Poisson regression models were undertaken to impute annual gender and age-specific (18–39 years, 40–69 years, ≥70 years) rates of diagnosed diabetes and to assess trends over time. The dependent variable was the number of cases of diagnosed diabetes and the exposure variables were year of data collection and age group. An interaction term between calendar year and age group was considered to explore whether the rates of change over time differed across age groups; a non-significant interaction indicated a common linear trend in prevalence. The predict command was used post analysis to calculate the expected rates of diagnosed diabetes for each calendar year of the study. The gender and age-specific predicted rates were applied to 2004–2015 population data so the absolute number of diabetes cases could be obtained. Annual population estimates were obtained from the Central Statistics Office (CSO), Ireland [21]. A census took place in Ireland in 2002, 2006 and 2011; data for other study years were CSO inter-censal estimates [21]. Prevalence was calculated by dividing the number of expected cases of doctor diagnosis of diabetes by the total study population and was expressed as a percentage with 95 % CI. Prevalence estimates were presented graphically in Excel.

## Results

### Study selection

**Fig. 1:**
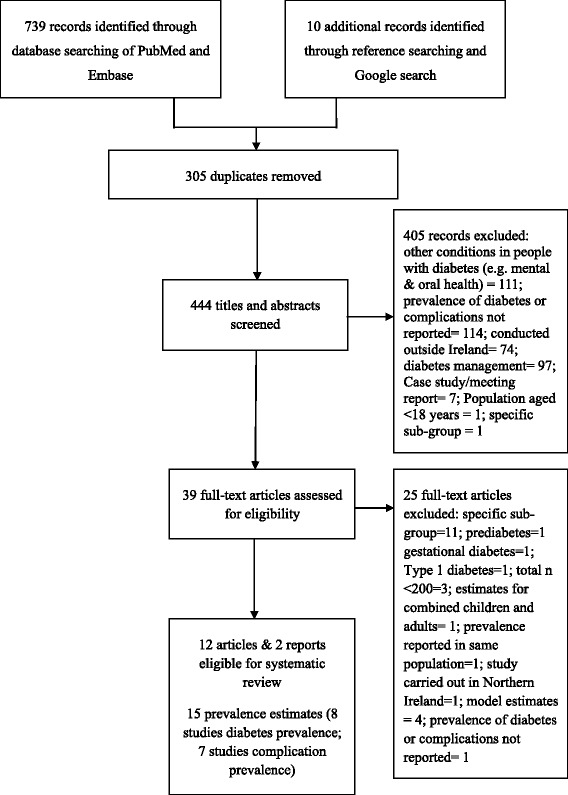
PRISMA flow chart depicting the selection process of articles included in the systematic review

Results of the literature search and the selection process are summarised in Fig. 1. One report [22] provided two estimates for diabetes prevalence from two separate studies [16, 17]. In total, 15 studies were eligible for inclusion; eight reporting estimates on diabetes prevalence and seven reporting estimates on complication prevalence. Of the included studies, the methodological quality was considered moderate in nine studies and high in the remaining studies (Additional file 2).

### Characteristics of selected studies

Characteristics of studies that reported the prevalence of diabetes or diabetes complications are presented in Tables 1 and 2. In all included studies, data collection were carried out between 1998 and 2011. Studies varied in terms of the study design, setting (national vs. regional), sampling approach and study quality. Of the 8 studies reporting on diabetes prevalence (Table 1), five articles had been published in peer-reviewed journals [11, 23–26], while three estimates were reported in two national reports [22, 27]. Of the 7 studies reporting diabetes complications (Table 2), six had been published in peer-reviewed journals [28–33], while one audit [34] provided data on the prevalence of diabetes related complications. Five studies utilised an objective data source to ascertain the prevalence of complications [28–30, 33, 34]. The diagnostic criteria for complications was unclear in three studies [31, 31, 34] whereas the remaining four used validated diagnostic criteria to identify cases [28–30, 33], however these criteria differed between studies reporting on the same complication.

**Table 1:** Characteristics of studies reporting the prevalence of diabetes or related complications among adults in the Republic of Ireland, 1998–2011

Author	Year of data collection	Study design	National or regional	Setting	Population	Sampling frame	Sampling method	Sample size	Males (%)	Age (years)	Study quality (out of 9)
Diabetes prevalence											
Sheily and Kelleher [22]	1998	Cross-sectional	National	Household	General population	Electoral register	Multistage sample	1632	47.7	≥55	7
Creagh et al. [23]	1998	Cross-sectional	Regional	17 GP practices	Primary Care Patients	Practice list	Stratified random	1018	48.2	50–69	6
Census Statistic Office (CSO) [27]	2001	Survey	National	Household	General population	Census	Total sample	3917203	-	≥18	5
Sheily and Kelleher [22]	2002	Cross-sectional	National	Household	General population	Electoral register	Multistage sample	1745	41.7	≥ 55	7
Balanda et al. [11]	2007	Cross-sectional	National	Household	General population	Geodirectory	Multistage probability	10,364	49.5	≥18	8
Gallagher et al. [24]	2009-2010	Cross-sectional	National	Database	Patients covered by GMS, LTI, DPS schemes	HSE-PCRS pharmacy claims data base	Total sample 2009 2010	3493974 3490877	-	≥16	6
Leahy et al. [25]	2009-2011	Cross-sectional analysis of longitudinal study	National	Household & designated health centre	General population	Geodirectory	Multi-stage probability	5377	46.5	≥50	8
OConnor et al. [26]	2010-2011	Cross-sectional	Regional	Primary care centre	Patients	Practice list	Random	2047	49.2	50–69	8

**Table 2:** Characteristics of studies reporting the prevalence of diabetes or complications among adults in the Republic of Ireland, 1998-2011

Author	Year of data collection	Study design	National or regional	Setting	Population	Sampling frame	Sampling method	Sample size	Males (%)	Age (years)	Study quality (out of 9)
Complication prevalence											
Kelliher et al. [28]	2003	Cross-sectional	National	National Council for Blind Ireland (NCBI)	All person registered blind	NCBI database	Total sample	6826	-	Adults	8
Buckley et al. [29]	2009	Cross-sectional	National	Population	People with diabetes	Hospital In-Patient Enquiry (HIPE) dataset	Total sample	723551	-	≥20 years	9
Marsden et al. [34]	2008-2009	Audit	Regional	20 general practices	Patients with T1 & T2 DM registered with diabetes structure care programme	Practice patient list	Every second person from list	1071	51.9	63 (sd 13)	5
Hurley et al. [30]	2008-2009	Cross-sectional analysis of longitudinal study	Regional	General practices with diabetes nurse	Patients with T1 & T2 DM	Practice diabetes register	Researchers selected eligible participants	563	60	64 (sd 13.4)	6
Farrell & Moran [31]	2010	Cross-sectional	Regional	30 general practices	T2 DM	Diabetes imitative database	Stratified sampling	309	-	-	5
Tracey et al. [32]	2009-2011	Cross-sectional analysis of longitudinal study	National	Household	General population	Geodirectory	Multi-stage probability	8175	53	≥50	8
McHugh et al. [33]	2011	Cross-sectional	Regional	30 general practices	Patients with T1 & T2 DM	Practice patient list	All persons with T1&T2DM invited	1542	57.3	65 (sd 13)	7

### Prevalence of diabetes in included studies

**Fig. 2:**
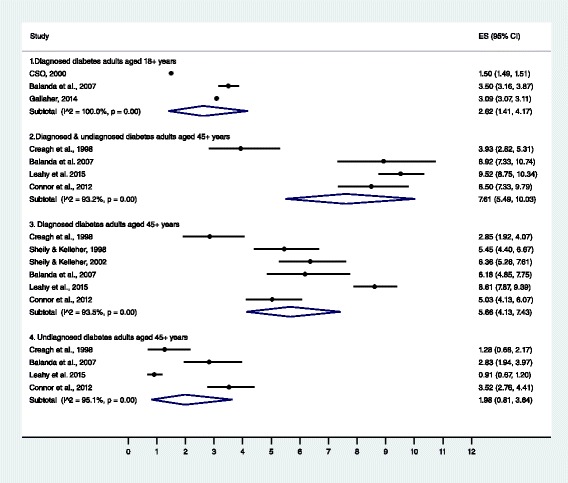
Forest plot of individual and summary diabetes prevalence estimates of included studies

Table 3 reports the prevalence of diabetes by study. Individual and summary estimates, based on a random-effects meta-analysis are illustrated in Fig. 2. There was significant heterogeneity in all groups. Sensitivity analysis only showed lower heterogeneity in combined prevalence rates for undiagnosed and diagnosed diabetes among adults aged over 45 years (I^2^ ≥ 25 %, *p* = 0.36); with a pooled prevalence of 9.2 % (95 % CI: 8.6–9.8) (Additional file 3). According to the Egger’s test, there was no evidence of publication bias (*p* = 0.27)*.*

**Table 3:** Prevalence of diabetes among adults in included studies, 1998-2011

Study	Year of data collection	Response rate (%)	Sample size	Age	Diabetes type	Diagnostic criteria	Estimate	Prevalence % (95 % CI)		
								Males	Females	Total
Sheily and Kelleher [22]	1998	62	1632	≥ 55 years	All	SR^a^	Diagnosed	6.1	4.3	5.4
Creagh et al. [23]	1998	69.1	1018	50–69 years	2	FPG^b^	Diagnosed Undiagnosed Total Total ≥65 years	- - - 13	- - - 7	2.8 1.2 3.9 (2.9–5.4) -
CSO [27]	July- Sept. 2001	-	3917203	≥ 18 years	All	SR	Diagnosed ≥18 years ≥65 years	- 1.7	- 1.4	1.5 4.5
Sheily and Kelleher [22]	2002	53	1745	≥ 55 years	All	SR	Diagnosed	8.0	5.1	6.4
Balanda et al. [11]	2007	62	10,364	≥ 18 years	All	SR or medication use or HbA1c^c^	Diagnosed 18–44 years 45+ years Total ≥ 18 years Undiagnosed (≥ 45 years) Total (diagnosed & undiagnosed ≥ 45 years)	- 6.8 (5.7–7.9) - 4.0 (1.6–6.3) 10.8 (8.2–13.4)	- 5.4 (4.3–6.6) - 1.7 (0.3–3.0) 7.1 (5.3–8.9)	0.7 (0.5–0.9) 6.1 (5.5–6.9) 3.5 (3.1–3.9) 2.8 (1.4–4.1) 8.9 (7.3–10.5)
Gallagher et al. [24]	2009 2010	-	3493974 3490877	≥ 18 years	2	At least 1 prescription of diabetes medication	Diagnosed 2009 2010	- -	- -	2.8 3.1
Leahy et al. [25]	2009–2011	62	5377	≥ 50 years	2	SR or medication use or HbA1c^c^	Diagnosed Undiagnosed Total (diagnosed & undiagnosed) 50–59 years 60–69 years 70–79 years 80+ years	- - 11.8 (10.3–13.3)* 5.1 (4.0–7.0) 6.0 (5.0–8.0) 12.0 (8.0–14.0) 10.0 (5.0–15.0)	- - 7.3 (6.0–8.5)* 4.0 14.0 (11.0–16.0) 17.0 (14.0–21.0) 25.0 (15.0–36.0)	8.6 (7.6–9.5) 0.9 (0.6–1.1) 9.5 (8.5–10.4) 5.0 (4.0–6.0) - - 16.0 (10.7–21.4)
OConnor et al. [26]	2010-2011	67.9	2047	50–69 years	2	SR or medication use or HbA1c^c^	Diagnosed Undiagnosed Total	6.8* 7.1* 11.1*	3.1* 2.7* 6.0*	5.0 (4.1–6.0) 3.5 (2.8–4.4) 8.5 (7.4–8.8)

*p for difference < 0.05

^a^ *SR* self-reported data; ^b^Fasting plasma glucose (American Diabetes Association criteria (ADA, 1997); ^c^HbA1c (ADA, 2010)

### Trends in the prevalence of diagnosed diabetes over time

In adults aged 18 years and over, the prevalence of diagnosed diabetes increased from 2.2 % (95 % CI: 1.7 %–2.7 %) in 1998 to 5.2 % (95 % CI: 5.1 %–5.3 %) in 2015 (*p*_trend_ = <0.001); representing an absolute mean increase of 0.17 % per year. In 2015, the incidence of diagnosed diabetes was 0.2/100 population.

**Fig. 3:**
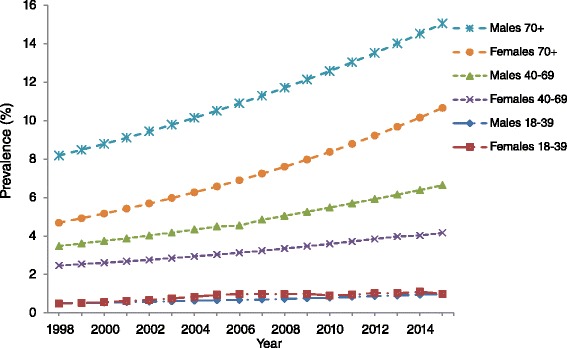
Prevalence of self-reported doctor diagnosed diabetes among adults in RoI, 1998–2015

Figure 3 illustrates the age-specific prevalence of self-reported diagnosed diabetes from 1998 to 2015. In adults aged between 18 and 39 years, the prevalence of self-reported doctor diagnosed diabetes remained stable between 1998 and 2015 in both men and women; *p*_trend_ >0.05. However, there was a significant increase in prevalence among men aged 40 to 69 years between 1998 (3.5 % [95 % CI: 3.4–3.6 %]) and 2015 (6.6 % [95 % CI: 6.5–6.7 %]; *p*_trend_ <0.001). The prevalence of diabetes also increased among women in the same age group over the same time period (1998–2.5 % [95 % CI: 2.4–2.5 %] to 2015- 4.2 % [95 % CI: 4.1–4.3 %]; *p*_trend_ <0.001). In those aged 70 years and over, an upward trend in prevalence among both men (1998–8.2 % [95 % CI: 8.0–8.3 %] to 2015- 15.1 % [95 % CI: 14.8–15.2 %]) and women (1998- 4.7 % [95 % CI: 4.5–4.8 %] to 2015- 10.7 % [95 % CI: 10.5–10.8 %]) was also observed; *p*_trend_ <0.001.

### Prevalence of microvascular and macrovascular complications

Table 4 describes the prevalence of microvascular and macrovascular complications in each included study. Five out of seven studies reported the prevalence of retinopathy [27, 29–31, 33]. Among people with type 2 diabetes, a population based study reported the prevalence of diabetic retinopathy to be 8.5 % in 2009–2011 [30]; a regional study, carried out among primary care patients, found a higher prevalence of 24.8 % [31]; however this estimate included patients with type 1 and 2 diabetes and was based on objective data. A similar estimate (25.6 %) was reported in a comparable cohort of primary care patients in a different region [33].

**Table 4:** Prevalence of microvascular and macrovascular complications in included studies, 2003–2011

Author	Year of study	Response rate (%)	Sample size	Age	Diabetes type	Time since diabetes diagnosis	Data source	Diagnostic method	Type of complication	Prevalence (%) Total
Kelliher et al. [28]	2003	-	6826	Adults	All	-	National blind registry	Visual acuity of <6/60 in better eye/visual field subtending angle of 20°/< less	Blindness due to diabetic retinopathy	4.7
Buckley et al. [29]	2009	-	723,551	≥20 years	All	-	Hospital discharge data	ICD-10 codes	Non-traumatic lower leg amputation	0.2
Marsden et al. [34]	Nov 2008-March 2009	72	1071	63 years (sd 13)	T1: 7.5 % T2: 92.3 %	15 years	Electronic & paper clinical notes & referral letters	- Risk classification score ACR 2.5–25 ACR >25 - - - -	Diabetic retinopathy Foot ulcer Microalbuminuria Proteinuria Myocardial Infarction Heart Failure Transient Ischemic Attack Stroke Total macrovascular	24.8 2.5 32.1 6.0 0.4 0.3 1.5 0.5 3.5
Hurley et al. [30]	Feb 2008- Sept 2009	68	563	64 years (sd 13.4)	T1: 10 % T2: 90 %	7.7 (8.2) years	Clinical foot examination & practice medical records	Scottish Intercollegiate Guidelines Network risk stratification system & previous doctor diagnosis	Documented diabetic neuropathy Foot ulceration Past amputation Neuropathy symptoms at examination	3.0 3.7 1.7 32
Farrell & Moran [31]	2010	-	309	-	T2	-	Chart review	-	Diabetic retinopathy Neuropathy Peripheral vascular disease Chronic kidney disease Cerebrovascular disease	6.5 12.3 12.9 5.5 5.2
Tracey et al. [32]	2009–2011	62	655	≥50 years	T2	5 (IQR 3–10) years	SR previous doctor diagnosis	-	Diabetic retinopathy Neuropathy Leg ulcer Nephropathy Proteinuria Total macrovascular	8.2 (6.2–10.9) 14.6 (11.4–18.2) 4.2 (2.8–6.4) 5.1 (3.4–7.6) 6.1 (4.3–8.6) 15.1 (12.2–18.4)
McHugh et al. [33]	2011	GP = 94 %; Screening uptake = 43 %	1542	65 years (sd 13)	T1: 4.9 % T2: 85.6 %	-	Eye examination & clinical records	Fundus 45° digital PASA-approved camera	Background (R1) Pre proliferative (R2) Proliferative (R3) Any diabetic retinopathy	21.5 (19.5–23.6) 3.4 (2.6–4.5) 0.7 (0.4–1.3) 25.6 (23.5–27.9)

In terms of diabetes-related neuropathy, a divergence in the reported prevalence between studies was also observed. Data from 12 primary care centres in the West of Ireland indicated a prevalence of past documented neuropathy to be 3 % [30]. On the other hand, a population-based study reported a prevalence of 14.6 % [32]. These patients had similar average duration since diagnosis (7.8 years [30] vs. 5.0 years [32]); however, the latter estimate was based on self-reported data. Prevalence rates for leg amputations were 1.7 % among primary care patients with diabetes [30]. In contrast, the prevalence of non-traumatic lower leg amputation was lower (0.2 %) in a population-based study which utilised national hospital discharge data [29].

With reference to nephropathy, prevalence among those with type 2 diabetes was similar in two studies [31, 32]. In the three studies presenting data on macrovascular complications, a marked difference in prevalence was observed. A primary care audit reported a prevalence of 3.5 % in patients with type 1 and 2 diabetes [34]. In contrast, among those with type 2 diabetes, a population based study reported a higher prevalence of 15.1 % [32].

## Discussion

This systematic review is the first study to compile all available evidence reporting the prevalence of diabetes (diagnosed and undiagnosed) and related complications (microvascular and macrovascular) among adults in Ireland between 1998 and 2015. Fifteen studies (eight describing diabetes prevalence and seven describing complication prevalence) were included.

Similar to other systematic reviews [35–37]; comparability between studies was limited due to differences in study population, sampling methods and diagnostic criteria. Additionally, substantial statistical heterogeneity was detected between studies reporting the prevalence of diabetes; therefore our pooled estimates have to be interpreted with caution. Sensitivity analysis, based on study quality, lowered the heterogeneity of combined prevalence rates for undiagnosed and diagnosed diabetes among adults aged over 45 years. However, this may reflect variability between prevalence estimates rather than study quality. Trends in diabetes prevalence could not be explored by meta-analysis, therefore, original data from four population-based national studies [16–19] were obtained to explore time trends in doctor diagnosed diabetes prevalence between 1998 and 2015. Over a seventeen year period, we observed an important increase in the national prevalence of self-reported diagnosed diabetes in Ireland.

Consistent with previous research [38–40] trends in the prevalence of self-reported diagnosed diabetes remained constant in adults aged 18 to 39 years, while an increasing prevalence was observed in the older age groups. We were unable to distinguish between the various types of diabetes in this review; however it can be assumed that type 2 diabetes is driving the increase in prevalence as it accounts for 90 % of all diabetes cases [1, 2]. The prevalence of diabetes was consistently higher in males compared to females. Evidence suggests that men are at a higher risk of developing type 2 diabetes as they develop diabetes at a lower BMI, are more predisposed to central fat deposition and are more prone to insulin resistance [41]. Therefore, men are more likely to develop type 2 diabetes in response to increasing levels of obesity [42]. On the other hand, the higher prevalence in the male population may reflect preferences in diagnostic methods. Evidence has highlighted that the prevalence of FPG diagnosed diabetes is higher among men, whereas women are more commonly diagnosed by a 2-h plasma glucose test [43]. While it is not possible to determine the method of diabetes diagnosis in this review; it is important to consider how these gender differences may influence diagnosed diabetes prevalence estimates over time.

Similar to diagnosed diabetes, trends in the prevalence of undiagnosed diabetes could not be explored by meta-analysis as only two nationally representative studies had relevant data [11, 25]. The prevalence of undiagnosed diabetes, based on HbA1c, decreased from 2.8 % in 2007 to 0.9 % in 2009–2011 among adults aged ≥45 years and ≥50 years, respectively. While the prevalence of diagnosed diabetes increased from 6.1 % in 2007 [11] to 8.6 % in 2009–2011 [25]. This shift from undiagnosed to diagnosed diabetes prevalence has also been observed in a study carried out in Germany [10]. This decrease in undiagnosed diabetes prevalence may be attributable to earlier detection of diabetes [10]. In Ireland, screening high risk patients for type 2 diabetes has been encouraged since the introduction of national guidelines for diabetes-care in 2002 [44]. Another study based on 29144 adults aged 45–75 years with private health insurance, reported the prevalence of undiagnosed diabetes to be 1.8 % in 2009–2012 [45]. However this estimate was derived from FPG; evidence suggests that the use of HbA1c may underestimate diabetes prevalence compared with estimates using FPG [38, 43, 46].

The prevalence of diabetes complications varied substantially between studies therefore comparisons between studies have to be interpreted with caution. These variations may be attributable to differences in disease duration or study population (type 1 and type 2 diabetes vs. type 2 diabetes), study setting (primary care vs. population-based) or heterogeneity in the criteria used to diagnose macrovascular and microvascular complications. Objective data describing the national prevalence of diabetic retinopathy was not available however, regional data on diabetic retinopathy showed that approximately 25 % of primary care patients with type 1 and type 2 diabetes had been diagnosed with this condition [33, 34]. This estimate is higher than a previous hospital-based study based on patients with type 2 diabetes (14.8 %) [47] and primary care data from the UK (19.6 %) [48] but lower than global prevalence estimates (34.6 %) [49]. Though, caution has to be applied when interpreting the results as both regional studies included in this review reported a low uptake rate of retinopathy screening at approximately 50 % [33, 34]. Additionally, characteristics between attenders and non-attenders were not compared in either study; hence it is possible that there were systematic differences between the two groups. Healthier people are more likely to participate in research; therefore the prevalence of diabetic retinopathy may have been underestimated. As a national screening programme for diabetic retinopathy was introduced in 2013 [50], future estimates based on this national programme may be more reliable.

### Limitations

The strengths and limitations of this systematic review should be noted. Both peer-reviewed articles and estimates detailed in the grey literature were included to limit the impact of publication bias. Original data from four national studies were obtained so trends in diagnosed diabetes prevalence could be examined over a 17 year period. Although response rates were below the optimal rate of 70 %, the representativeness of each study has been demonstrated previously [18, 51], so it can be assumed that the results presented can be generalised to the Irish population.

However, several limitations need to be acknowledged. Firstly, studies included in this review were of moderate to high quality; however, six of the included studies relied on self-reporting to determine the prevalence of diagnosed diabetes and one study relied on self-reporting to determine the prevalence of diabetes related complications. This approach is prone to misclassification bias which can result in an inaccurate estimation of prevalence [52]. When compared to medical records, data from self-report have been shown to underestimate the prevalence of diabetic retinopathy [53]. However, moderate to high levels of agreement between diabetes prevalence and self-report have been shown in several studies [54–56]. Although only data on self-reported diabetes were available, results from trend analysis are in line with other developed countries. Secondly, without the inclusion of undiagnosed diabetes in our trend analysis, we acknowledge that diabetes prevalence is underestimated. Finally, significant increases in diagnosed diabetes prevalence were observed over time but these increases may be attributed to heightened awareness among patients, changes in clinical practices, including increased screening for type 2 diabetes, and better survival rates for patients with diabetes [57]. However, there is a lack of data on mortality rates among people with diabetes in Ireland; therefore it is not possible to determine whether our increasing trends in prevalence are due to improved health outcomes in those with diabetes.

## Conclusion

This review provides the first comprehensive overview of the burden of diabetes in Ireland. In the absence of a national diabetes register, the findings in this review provide a robust estimate of the trends in prevalence of doctor diagnosed diabetes among the adult population in Ireland. Findings from this review are in accordance with the Euro Diabetes Index (2014) [12]; there is a lack of information relating to the prevalence of undiagnosed diabetes, macrovascular and microvascular complications. Interpretation of available data was limited due to inconsistencies in reporting, limited availability of objective data and standardisation in diagnostic criteria. We suggest that the true burden of diabetes in Ireland is underestimated [58]. In 2010, the National Clinical Programme in Diabetes was established to improve and standardise patient care in Ireland [59]. Reliable baseline data are needed to monitor improvements in care over time at a national level. Therefore, we suggest that a comprehensive national diabetes register is urgently needed in Ireland.

## Additional files

Additional file 1: **Electronic search strategies for articles.** (DOCX 11 kb)

Additional file 2: **Critical appraisal checklist for studies reporting prevalence data.** (DOCX 25 kb)

Additional file 3: **Sensitivity analysis based on high quality studies.** (DOCX 16 kb)
