# Supplementary material for: Epidemiology of diabetes and complications among adults in the Republic of Ireland 1998-2015: a systematic review and meta-analysis
Source: BMC Public Health. 2016 Feb 9;16:132. doi: 10.1186/s12889-016-2818-2 (PMC4748605; doi:10.1186/s12889-016-2818-2)
Supplement: Supplementary file 1 — Electronic search strategies for articles. (DOCX 11 kb) [file 12889_2016_2818_MOESM1_ESM.docx]

Marsha Tracey et al

Supplementary file 1

Electronic search strategies for articles

**PUBMED**

1. Diabetes mellitus (MeSH)

2. Diabet*

3. 1 or 2

4. Prevalence (MeSH)

5. Ireland (MeSH)

6. 3 and 4 and 5

7. Journal Article (Article type)

8. Observational Study (Article type)

9. Systematic review (Article type)

10. Humans (Species)

11. English (Language)

**Search #1:** 1^st^ January 1998- 31^st^ December 2013 (Publication dates)

**Search #2:** 1^st^ January 2014- 30^th^ November 2015 (Publication dates)

#1 Results: 143

#2 Results: 10

**Embase**

1. 'diabetes mellitus'/exp

2. diabet*

3. 1 or 2

4. 'Ireland'/exp

5. ‘Prevalence’/exp

**Search #1**: 3 and 4 and 5 (Limits: ([article]/lim OR [article in press]/lim) AND ([young adult]/lim OR [adult]/lim OR [middle aged]/lim OR [aged]/lim OR [very elderly]/lim) AND [humans]/lim AND [english]/lim AND [embase]/lim AND [1998-2014]/py

**Search #2**: 3 and 4 and 5 (Limits: ([article]/lim OR [article in press]/lim) AND ([young adult]/lim OR [adult]/lim OR [middle aged]/lim OR [aged]/lim OR [very elderly]/lim) AND [humans]/lim AND [english]/lim AND [embase]/lim AND [2014-2015]/py

#1 Results: 555

#2 Results: 31
