# Supplementary material for: Epidemiology of diabetes and complications among adults in the Republic of Ireland 1998-2015: a systematic review and meta-analysis
Source: BMC Public Health. 2016 Feb 9;16:132. doi: 10.1186/s12889-016-2818-2 (PMC4748605; doi:10.1186/s12889-016-2818-2)
Supplement: Supplementary file 2 — Critical appraisal checklist for studies reporting prevalence data. (DOCX 25 kb) [file 12889_2016_2818_MOESM2_ESM.docx]

Marsha Tracey et al

Supplementary file 2

Critical appraisal checklist for studies reporting prevalence data (12)

SLÁN 1998: Sheily et al. (2004)

| Methodological criteria | Yes | No | Unclear | Not applicable | Score |
| --- | --- | --- | --- | --- | --- |
| 1. Was the sample representative of the target population? | X |  |  |  | 1 |
| 2. Were study participants recruited in an appropriate way? | X |  |  |  | 1 |
| 3. Was the sample size adequate? | X |  |  |  | 1 |
| 4. Were the study subjects and setting described in detail? | X |  |  |  | 1 |
| 5. Is the data analysis conducted with sufficient coverage of the identified sample? |  | X |  |  | 0 |
| 6. Were objective, standard criteria used for measurement of the condition? |  | X |  |  | 0 |
| 7. Was the condition measured reliably? | X |  |  |  | 1 |
| 8. Was there appropriate statistical analysis? | X |  |  |  | 1 |
| 9. Are all important confounding factors/subgroups/ differences identified and accounted for? | X |  |  |  | 1 |
| 10. Were subpopulations identified using objective criteria? |  |  |  | X |  |
| Total score |  |  |  |  | 7/9 |

Creagh et al (2002)

| Methodological criteria | Yes | No | Unclear | Not applicable | Score |
| --- | --- | --- | --- | --- | --- |
| 1. Was the sample representative of the target population? | X |  |  |  | 1 |
| 2. Were study participants recruited in an appropriate way? | X |  |  |  | 1 |
| 3. Was the sample size adequate? | X |  |  |  | 1 |
| 4. Were the study subjects and setting described in detail? | X |  |  |  | 1 |
| 5. Is the data analysis conducted with sufficient coverage of the identified sample? | X |  |  |  | 1 |
| 6. Were objective, standard criteria used for measurement of the condition? | X |  |  |  | 1 |
| 7. Was the condition measured reliably? |  |  | X |  | 0 |
| 8. Was there appropriate statistical analysis? |  |  | X |  | 0 |
| 9. Are all important confounding factors/subgroups/ differences identified and accounted for? | X |  |  |  | 1 |
| 10. Were subpopulations identified using objective criteria? |  |  |  | X |  |
| Total score |  |  |  |  | 7/9 |

Central Statistics Office (2001)

| Methodological criteria | Yes | No | Unclear | Not applicable | Score |
| --- | --- | --- | --- | --- | --- |
| 1. Was the sample representative of the target population? | X |  |  |  | 1 |
| 2. Were study participants recruited in an appropriate way? | X |  |  |  | 1 |
| 3. Was the sample size adequate? | X |  |  |  | 1 |
| 4. Were the study subjects and setting described in detail? | X |  |  |  | 1 |
| 5. Is the data analysis conducted with sufficient coverage of the identified sample? |  |  | X |  | 0 |
| 6. Were objective, standard criteria used for measurement of the condition? |  | X |  |  | 0 |
| 7. Was the condition measured reliably? | X |  |  |  | 1 |
| 8. Was there appropriate statistical analysis? |  |  | X |  | 0 |
| 9. Are all important confounding factors/subgroups/ differences identified and accounted for? |  | X |  |  | 0 |
| 10. Were subpopulations identified using objective criteria? |  |  |  | X |  |
| Total score |  |  |  |  | 5/9 |

SLÁN 2002: Sheily et al. (2004)

| Methodological criteria | Yes | No | Unclear | Not applicable | Score |
| --- | --- | --- | --- | --- | --- |
| 1. Was the sample representative of the target population? | X |  |  |  | 1 |
| 2. Were study participants recruited in an appropriate way? | X |  |  |  | 1 |
| 3. Was the sample size adequate? | X |  |  |  | 1 |
| 4. Were the study subjects and setting described in detail? | X |  |  |  | 1 |
| 5. Is the data analysis conducted with sufficient coverage of the identified sample? |  | X |  |  | 0 |
| 6. Were objective, standard criteria used for measurement of the condition? |  | X |  |  | 0 |
| 7. Was the condition measured reliably? | X |  |  |  | 1 |
| 8. Was there appropriate statistical analysis? | X |  |  |  | 1 |
| 9. Are all important confounding factors/subgroups/ differences identified and accounted for? | X |  |  |  | 1 |
| 10. Were subpopulations identified using objective criteria? |  |  |  | X |  |
| Total score |  |  |  |  | 7/9 |

Balanda et al. (2013)

| Methodological criteria | Yes | No | Unclear | Not applicable | Score |
| --- | --- | --- | --- | --- | --- |
| 1. Was the sample representative of the target population? | X |  |  |  | 1 |
| 2. Were study participants recruited in an appropriate way? | X |  |  |  | 1 |
| 3. Was the sample size adequate? | X |  |  |  | 1 |
| 4. Were the study subjects and setting described in detail? | X |  |  |  | 1 |
| 5. Is the data analysis conducted with sufficient coverage of the identified sample? | X |  |  |  | 0 |
| 6. Were objective, standard criteria used for measurement of the condition? |  | X |  |  | 0 |
| 7. Was the condition measured reliably? | X |  |  |  | 1 |
| 8. Was there appropriate statistical analysis? | X |  |  |  | 1 |
| 9. Are all important confounding factors/subgroups/ differences identified and accounted for? | X |  |  |  | 1 |
| 10. Were subpopulations identified using objective criteria? |  |  |  | X |  |
| Total score |  |  |  |  | 8/9 |

Leahy et al. (2015)

| Methodological criteria | Yes | No | Unclear | Not applicable | Score |
| --- | --- | --- | --- | --- | --- |
| 1. Was the sample representative of the target population? | X |  |  |  | 1 |
| 2. Were study participants recruited in an appropriate way? | X |  |  |  | 1 |
| 3. Was the sample size adequate? | X |  |  |  | 1 |
| 4. Were the study subjects and setting described in detail? | X |  |  |  | 1 |
| 5. Is the data analysis conducted with sufficient coverage of the identified sample? | X |  |  |  | 1 |
| 6. Were objective, standard criteria used for measurement of the condition? |  | X |  |  | 0 |
| 7. Was the condition measured reliably? | X |  |  |  | 1 |
| 8. Was there appropriate statistical analysis? | X |  |  |  | 1 |
| 9. Are all important confounding factors/subgroups/ differences identified and accounted for? | X |  |  |  | 1 |
| 10. Were subpopulations identified using objective criteria? |  |  |  | X |  |
| Total score |  |  |  |  | 8/9 |

Gallagher et al. (2014)

| Methodological criteria | Yes | No | Unclear | Not applicable | Score |
| --- | --- | --- | --- | --- | --- |
| 1. Was the sample representative of the target population? | X |  |  |  | 1 |
| 2. Were study participants recruited in an appropriate way? | X |  |  |  | 1 |
| 3. Was the sample size adequate? | X |  |  |  | 1 |
| 4. Were the study subjects and setting described in detail? |  | X |  |  | 0 |
| 5. Is the data analysis conducted with sufficient coverage of the identified sample? | X |  |  |  | 1 |
| 6. Were objective, standard criteria used for measurement of the condition? | X |  |  |  | 1 |
| 7. Was the condition measured reliably? |  | X |  |  | 0 |
| 8. Was there appropriate statistical analysis? | X |  |  |  | 1 |
| 9. Are all important confounding factors/subgroups/ differences identified and accounted for? |  | X |  |  | 0 |
| 10. Were subpopulations identified using objective criteria? |  |  |  | X |  |
| Total score |  |  |  |  | 6/9 |

O’Connor et al (2013)

| Methodological criteria | Yes | No | Unclear | Not applicable | Score |
| --- | --- | --- | --- | --- | --- |
| 1. Was the sample representative of the target population? | X |  |  |  | 1 |
| 2. Were study participants recruited in an appropriate way? | X |  |  |  | 1 |
| 3. Was the sample size adequate? | X |  |  |  | 1 |
| 4. Were the study subjects and setting described in detail? | X |  |  |  | 1 |
| 5. Is the data analysis conducted with sufficient coverage of the identified sample? | X |  |  |  | 1 |
| 6. Were objective, standard criteria used for measurement of the condition? |  | X |  |  | 0 |
| 7. Was the condition measured reliably? | X |  |  |  | 1 |
| 8. Was there appropriate statistical analysis? | X |  |  |  | 1 |
| 9. Are all important confounding factors/subgroups/ differences identified and accounted for? | X |  |  |  | 1 |
| 10. Were subpopulations identified using objective criteria? |  |  |  | X |  |
| Total score |  |  |  |  | 8/9 |

Kelliher et al. (2006)

| Methodological criteria | Yes | No | Unclear | Not applicable | Score |
| --- | --- | --- | --- | --- | --- |
| 1. Was the sample representative of the target population? | X |  |  |  | 1 |
| 2. Were study participants recruited in an appropriate way? | X |  |  |  | 1 |
| 3. Was the sample size adequate? | X |  |  |  | 1 |
| 4. Were the study subjects and setting described in detail? | X |  |  |  | 1 |
| 5. Is the data analysis conducted with sufficient coverage of the identified sample? | X |  |  |  | 1 |
| 6. Were objective, standard criteria used for measurement of the condition? | X |  |  |  | 1 |
| 7. Was the condition measured reliably? | X |  |  |  | 1 |
| 8. Was there appropriate statistical analysis? | X |  |  |  | 1 |
| 9. Are all important confounding factors/subgroups/ differences identified and accounted for? |  | X |  |  | 0 |
| 10. Were subpopulations identified using objective criteria? |  |  |  | X |  |
| Total score |  |  |  |  | 8/9 |

Buckley et al (2012)

| Methodological criteria | Yes | No | Unclear | Not applicable | Score |
| --- | --- | --- | --- | --- | --- |
| 1. Was the sample representative of the target population? | X |  |  |  | 1 |
| 2. Were study participants recruited in an appropriate way? | X |  |  |  | 1 |
| 3. Was the sample size adequate? | X |  |  |  | 1 |
| 4. Were the study subjects and setting described in detail? | X |  |  |  | 1 |
| 5. Is the data analysis conducted with sufficient coverage of the identified sample? | X |  |  |  | 1 |
| 6. Were objective, standard criteria used for measurement of the condition? | X |  |  |  | 1 |
| 7. Was the condition measured reliably? | X |  |  |  | 1 |
| 8. Was there appropriate statistical analysis? | X |  |  |  | 1 |
| 9. Are all important confounding factors/subgroups/ differences identified and accounted for? | X |  |  |  | 1 |
| 10. Were subpopulations identified using objective criteria? |  |  |  | X |  |
| Total score |  |  |  |  | 9/9 |

Marsden et al (2010)

| Methodological criteria | Yes | No | Unclear | Not applicable | Score |
| --- | --- | --- | --- | --- | --- |
| 1. Was the sample representative of the target population? | X |  |  |  | 1 |
| 2. Were study participants recruited in an appropriate way? |  | X |  |  | 0 |
| 3. Was the sample size adequate? |  | X |  |  | 0 |
| 4. Were the study subjects and setting described in detail? | X |  |  |  | 1 |
| 5. Is the data analysis conducted with sufficient coverage of the identified sample? |  |  | X |  | 0 |
| 6. Were objective, standard criteria used for measurement of the condition? |  |  | X |  | 0 |
| 7. Was the condition measured reliably? | X |  |  |  | 1 |
| 8. Was there appropriate statistical analysis? | X |  |  |  | 1 |
| 9. Are all important confounding factors/subgroups/ differences identified and accounted for? | X |  |  |  | 1 |
| 10. Were subpopulations identified using objective criteria? |  |  |  | X |  |
| Total score |  |  |  |  | 5/9 |

Hurley et al. (2013)

| Methodological criteria | Yes | No | Unclear | Not applicable | Score |
| --- | --- | --- | --- | --- | --- |
| 1. Was the sample representative of the target population? | X |  |  |  | 1 |
| 2. Were study participants recruited in an appropriate way? |  | X |  |  | 0 |
| 3. Was the sample size adequate? |  |  | X |  | 0 |
| 4. Were the study subjects and setting described in detail? | X |  |  |  | 1 |
| 5. Is the data analysis conducted with sufficient coverage of the identified sample? |  | X |  |  | 0 |
| 6. Were objective, standard criteria used for measurement of the condition? | X |  |  |  | 0 |
| 7. Was the condition measured reliably? | X |  |  |  | 1 |
| 8. Was there appropriate statistical analysis? | X |  |  |  | 1 |
| 9. Are all important confounding factors/subgroups/ differences identified and accounted for? | X |  |  |  | 1 |
| 10. Were subpopulations identified using objective criteria? |  |  |  | X |  |
| Total score |  |  |  |  | 5/9 |

Mc Hugh et al. (2013)

| Methodological criteria | Yes | No | Unclear | Not applicable | Score |
| --- | --- | --- | --- | --- | --- |
| 1. Was the sample representative of the target population? | X |  |  |  | 1 |
| 2. Were study participants recruited in an appropriate way? | X |  |  |  | 1 |
| 3. Was the sample size adequate? |  |  | X |  | 0 |
| 4. Were the study subjects and setting described in detail? | X |  |  |  | 1 |
| 5. Is the data analysis conducted with sufficient coverage of the identified sample? | X |  |  |  | 1 |
| 6. Were objective, standard criteria used for measurement of the condition? | X |  |  |  | 1 |
| 7. Was the condition measured reliably? | X |  |  |  | 1 |
| 8. Was there appropriate statistical analysis? | X |  |  |  | 1 |
| 9. Are all important confounding factors/subgroups/ differences identified and accounted for? |  | X |  |  | 0 |
| 10. Were subpopulations identified using objective criteria? |  |  |  | X |  |
| Total score |  |  |  |  | 7/9 |

Farrell & Moran, (2014)

| Methodological criteria | Yes | No | Unclear | Not applicable | Score |
| --- | --- | --- | --- | --- | --- |
| 1. Was the sample representative of the target population? |  |  | X |  | 0 |
| 2. Were study participants recruited in an appropriate way? | X |  |  |  | 1 |
| 3. Was the sample size adequate? |  |  | X |  | 0 |
| 4. Were the study subjects and setting described in detail? | X |  |  |  | 1 |
| 5. Is the data analysis conducted with sufficient coverage of the identified sample? | X |  |  |  | 1 |
| 6. Were objective, standard criteria used for measurement of the condition? |  |  | X |  | 0 |
| 7. Was the condition measured reliably? | X |  |  |  | 1 |
| 8. Was there appropriate statistical analysis? | X |  |  |  | 1 |
| 9. Are all important confounding factors/subgroups/ differences identified and accounted for? |  | X |  |  | 0 |
| 10. Were subpopulations identified using objective criteria? |  |  |  | X |  |
| Total score |  |  |  |  | 5/9 |

Tracey et al., (2015)

| Methodological criteria | Yes | No | Unclear | Not applicable | Score |
| --- | --- | --- | --- | --- | --- |
| 1. Was the sample representative of the target population? | X |  |  |  | 1 |
| 2. Were study participants recruited in an appropriate way? | X |  |  |  | 1 |
| 3. Was the sample size adequate? | X |  |  |  | 1 |
| 4. Were the study subjects and setting described in detail? | X |  |  |  | 1 |
| 5. Is the data analysis conducted with sufficient coverage of the identified sample? | X |  |  |  | 1 |
| 6. Were objective, standard criteria used for measurement of the condition? |  | X |  |  | 0 |
| 7. Was the condition measured reliably? | X |  |  |  | 1 |
| 8. Was there appropriate statistical analysis? | X |  |  |  | 1 |
| 9. Are all important confounding factors/subgroups/ differences identified and accounted for? | X |  |  |  | 1 |
| 10. Were subpopulations identified using objective criteria? |  |  |  | X |  |
| Total score |  |  |  |  | 8/9 |
