# Supplementary material for: Epidemiology of diabetes and complications among adults in the Republic of Ireland 1998-2015: a systematic review and meta-analysis
Source: BMC Public Health. 2016 Feb 9;16:132. doi: 10.1186/s12889-016-2818-2 (PMC4748605; doi:10.1186/s12889-016-2818-2)
Supplement: Supplementary file 3 — Sensitivity analysis based on high quality studies. (DOCX 16 kb) [file 12889_2016_2818_MOESM3_ESM.docx]

Marsha Tracey et al.

Supplementary file 3

Sensitivity analysis based on high quality studies
